# Medical Complexity of Children with Special Healthcare Needs and Healthcare Experiences

**DOI:** 10.3390/children11070775

**Published:** 2024-06-27

**Authors:** Hye-Jung Yun, M. L. Parker, Cynthia B. Wilson, Ming Cui

**Affiliations:** 1 The Florida Center for Prevention Research, Florida State University, Tallahassee, FL 32301, USA; hyun@fsu.edu (H.-J.Y.); cbwilson2@fsu.edu (C.B.W.); 2 School of Education and Human Development, Fairfield University, Fairfield, CT 06824, USA; mparker@fairfield.edu; 3 Department of Human Development and Family Science, Florida State University, Tallahassee, FL 32306, USA

**Keywords:** care coordination (CC), children with special healthcare needs (CSHCN), family-centered care (FCC), medical complexity, shared decision-making (SDM)

## Abstract

The rising prevalence of CSHCN has led to significant challenges for caregivers, particularly mothers, who face difficulties from caregiving demands and managing complex healthcare interactions. The objective of this study was to examine the association between the medical complexity of CSHCN and the healthcare experiences of their mothers while exploring the influence of sociodemographic factors on these associations. The study utilized data from the 2016–2020 National Survey of Children’s Health (NSCH), involving 17,434 mothers of CSHCN. Mothers provided information on the medical complexity of CSHCN, healthcare experiences (care coordination, family-centered care, and shared decision-making), and sociodemographic information (race, community, insurance, child sex, age, and federal poverty level). Results from multiple regressions revealed that greater medical complexity was associated with more negative healthcare experiences. Minoritized mothers, those in rural areas, and families with lower income reported lower levels of family-centered care, indicating significant disparities. Additionally, the negative association between medical complexity and healthcare experiences was pronounced for White families and those with private insurance compared to minoritized families and those with public insurance. This study highlights the necessity for targeted interventions to improve care coordination, family-centered care, and shared decision-making, emphasizing the need for a comprehensive, family-centered approach to address healthcare disparities and promote health equity for CSHCN and their families.

## 1. Introduction

Addressing and advocating for the well-being of children with chronic and complex medical, developmental, or behavioral conditions is a public health imperative. According to the National Survey of Children’s Health (NSCH), approximately 18.5% of children under 18 have special healthcare needs, representing a 6% increase since 2001 [1]. The increased prevalence of children with special healthcare needs (CSHCN) over the past two decades is partially attributed to advancements in medical technology and improved diagnostic capabilities for developmental disorders [2]. The growing rate of CSHCN also leads to an expanding disparity between the need for providers and the accessibility of services that meet the recommended standard of care [3]. A health equity framework highlights important factors that contribute to the growing disparities in care between CSHCN and children in the general population [4]. 

The increase in healthcare systems, coupled with challenges in accessing recommended treatments, often necessitates parents of CSHCN to assume a greater role in navigating and advocating for necessary healthcare services. Zablotsky et al. [5] highlighted this trend, emphasizing the declining accessibility of recommended treatments and the subsequent burden placed on parents to ensure their children receive appropriate care. Mothers often take more of the primary role in healthcare decision-making for their children, including coordinating medical appointments and administering treatments. Therefore, the goal of the present study was to understand the association between medical complexity and the healthcare experiences of mothers of CSHCN. Furthermore, Valeras [6] emphasized that meaningful improvements on both individual and population levels could only be achieved by examining and understanding the interrelationships among social and behavioral determinants of health, mental health, and physical health as integral components of a unified whole. Thus, we examined the roles of sociodemographic factors (i.e., race/ethnicity, community rurality, and insurance type) in CSHCN complexity and healthcare experiences. 

### 1.1. Medical Complexity of CSHCN

Children with special healthcare needs (CSHCN) are broadly characterized by at least one chronic health condition that cumulatively affects three or more organ systems, severely reduces cognitive or physical functioning, and requires the use of medication, equipment, therapy, surgery, or other treatments [1]. Medical complexity among CSHCN is often classified into two dimensions: the degree of functional limitations associated with the condition(s) and the need for specialized healthcare services [7]. More specifically, existing methods used to evaluate the degree of medical complexity may include the number of body systems affected by conditions (e.g., >2), progressive versus episodic conditions, or dependence on specific services/treatments such as technology [8]. Medical complexity significantly influences the healthcare experiences of CSHCN across multiple dimensions. Understanding the intricate interplay between medical complexity and healthcare experiences is essential for optimizing care delivery and improving outcomes for these vulnerable populations. 

### 1.2. Healthcare Experiences 

A natural byproduct of increased healthcare utilization is the addition of more providers (e.g., physicians and therapists) and organizations (e.g., hospitals and clinics) to the systems of care. Kenney and colleagues [9] explained that mothers of CSHCN reported lower family-provider partnerships and satisfaction with healthcare services than children without special healthcare needs. Children’s access to the necessary services and the quality of their care has significant influences on children and family outcomes, particularly among CSHCN with greater complexity [10]. The expansion of healthcare utilization in the care of CSHCN underscores the importance of effective care coordination, family-centered care, and shared decision-making in optimizing outcomes for both children and their families. 

Care Coordination (CC). The Council on Children with Disabilities and Medical Home Implementation Project Advisory Committee [11] advocates for CC in primary healthcare as a means of cutting across subspecialty divisions to improve healthcare delivery. Previous studies have consistently highlighted the positive impact of CC on meeting the diverse healthcare needs of CSHCN. For instance, a study by Kuo et al. [12] demonstrated that CC interventions significantly decreased unmet healthcare needs among children with chronic conditions. This finding was further supported by the work of McAllister et al. [13], which emphasized the role of CC in improving access to specialized services and reducing unmet needs in this population. 

Family-Centered Care (FCC). Family-centered health care is widely recognized as essential for CSHCN. The Council on Children with Disabilities and Medical Home Implementation Project Advisory Committee [11] endorses FCC as an integral part of the medical home model, particularly for CSHCN. The concept of FCC among CSHCN highlights the interrelatedness of the child’s treatment within the context of their families and communities [14]. This approach promotes a collaborative relationship between healthcare providers and family members to encourage family engagement in treatment decisions [11]. Research consistently demonstrates the positive effect of FCC on both children and families, such as fewer unmet healthcare needs, increased family functioning, and reduced healthcare expenses [15,16]. 

Shared Decision-Making (SDM). The emphasis on higher-quality healthcare at reduced costs has driven policy attention toward SDM. SDM involves collaborative decision-making between patients and clinicians in making healthcare choices, ensuring that patients are well-informed and engaged in decisions regarding their care. This approach recognizes patients’ rights to actively participate in their own healthcare and ensures that decisions align with their preferences and values within their circumstances [17]. Stacey et al. [18] found that patients who were provided with an SDM tool, such as Patient Decision Aids (PtDAs) or a conversation aid, reported a greater sense of involvement in the decision-making process.

### 1.3. Medical Complexity of CSHCN and Healthcare Experiences

According to family systems theory [19], families operate as interconnected systems, wherein the condition and behavior of one family member can significantly influence others and overall family dynamics. When a child has special healthcare needs, particularly those with greater medical complexity, family functioning and interactions are profoundly affected. Families of CSHCN need to navigate their child’s healthcare needs through collaboration with healthcare providers, emphasizing CC and SDM within a family-centered framework. In addition, parenting stress theory [20] suggests that the demands associated with caring for CSHCN can lead to heightened stress and negative experiences associated with the increasing demands of healthcare for parents. The increased medical complexity of CSHCN exacerbates these stressors, as parents are tasked with additional responsibilities such as accessing services, coordinating care, and managing various treatments. 

Empirical evidence generally supports these theoretical perspectives, indicating that greater medical complexity among CSHCN negatively impacts not only parents in general but particularly mothers who often bear the primary caregiving responsibilities. Studies have shown that increased medical complexity was associated with higher rates of delayed or foregone care and unmet healthcare needs reported by parents of CSHCN [21,22]. Specifically, mothers of medically complex CSHCN face numerous challenges, including difficulties in healthcare experiences and accessing necessary services [15]. Research by Hodgetts et al. [23] underscores the adverse impact of medical complexity on CC, revealing that mothers of medically complex CSHCN often encounter challenges in navigating the healthcare system and coordinating various aspects of their child’s care. Similarly, McAllister et al. [13] found that medical complexity was negatively associated with FCC, highlighting difficulties faced by mothers in engaging with healthcare providers and accessing comprehensive, family-centered services. Also, previous research indicated that the medical complexity of CSHCN could significantly hinder effective SDM [24]. Thus, this study aimed to explore the impact of medical complexity faced by mothers of CSHCN on various healthcare experiences including CC, FCC, and SDM to better support these families within a family-centered framework.

### 1.4. Health Equity Framework: Sociodemographic Factors

Resources are not equally accessible to all families due to existing disparities in health care. The existing inequities for certain groups of CSHCN amplify the barriers to healthcare associated with greater medical complexity. A health equity framework (HEF) offers a valuable lens to explore disparities in healthcare experiences among CSHCN and their parents. HEF highlights social determinants (e.g., sociodemographic factors) that contribute to the medical complexity of CSHCN and healthcare experience disparities among those at risk of poor health because of social conditions [4]. Recent estimates indicate child health equity in the U.S. has increased gradually over the past 20 years [25], yet CSHCN face additional healthcare barriers compared to children in the general population. Similarly, mothers of CSHCN are disproportionately affected by the additional caregiving demands resulting in negative healthcare experiences [26]. HEF specifically emphasizes sociodemographic influences that exacerbate the existing inequalities in health outcomes among CSHCN and their families. Among the identified social determinants that contribute to disparities in health, race/ethnicity, community setting, and insurance type have been shown to have direct implications for the medical complexity of CSHCN and healthcare among mothers. 

Compared with White children, CSHCN from racially minoritized families may experience more challenges implementing comprehensive CC initiatives and hindering the effectiveness of CC efforts, therefore leading to more negative healthcare experiences by mothers [27,28,29]. Certain FCC practices, such as parental partnership, time spent with the child, and sensitivity to family values have been shown to explain the unmet treatment needs among Black and Hispanic CSHCN [30]. Additionally, mothers of CSHCN living in rural communities experience difficulties with transportation and a growing shortage of healthcare providers (i.e., Healthcare Provider Shortage Areas), resulting in increased unmet healthcare needs [31,32]. Finally, although government-funded insurance policies (e.g., Medicaid) have become a prominent source of healthcare coverage for low-income children with chronic health problems, there continue to be inequities inherent within the program [33]. For example, underinsured families are less likely to receive FCC [34,35,36], thereby limiting the positive outcomes associated with FCC. Taken together, CSHCN from racially minoritized families, living in rural communities, and under-insured (e.g., solely relying on public health insurance such as Medicaid) may lack adequate healthcare infrastructure and resources, which often leads to negative healthcare experiences by parents, especially mothers of CSHCN.

### 1.5. The Present Study 

Given the importance of the well-being of CSHCN and their families, we aim to contribute to the field by applying several theories to examine the association between the medical complexity of CSHCN and the healthcare experiences of mothers. Specifically, we evaluate multiple dimensions of the healthcare experiences of mothers, including CC, FCC, and SDM. Based on family systems theory and parenting stress theory, we hypothesized that the medical complexity of CSHCN would be negatively related to the healthcare experiences of mothers of CSHCN (H1). We further identify critical sociodemographic factors that affect the medical complexity of CSHCN and healthcare experiences among mothers of CSHCN. We specifically evaluate the potential main and moderating roles of child race/ethnicity (minority vs. White), community setting (rural vs. non-rural), and insurance type (public vs. private) on the medical complexity of CSHCN and healthcare experiences (CC, FCC, and SDM). Based on the health equity framework [4], we hypothesized that CSHCN of minoritized status in rural communities and with public health insurance had more negative healthcare experiences by mothers (main effects, H2a) and that they were also more vulnerable to negative experiences from medical complexity (moderating effects, H2b). 

## 2. Methods

### 2.1. Data Source

Data used in the current study came from the 2016–2020 National Survey of Children’s Health (NSCH), which oversamples CSHCN. The NSCH is an annual cross-sectional survey conducted by the U.S. Census Bureau and sponsored by the U.S. Maternal and Child Health Bureau [37] to assess child and family health-related information among a nationally representative sample. Data were merged from the 2016 through 2020 surveys using the guidelines from the NSCH Guide to Multi-Year Analysis [38], resulting in the largest, nationally representative sample of CSHCN. Sampling weights, which are designed to account for both survey non-response and varying probabilities of selection, are included in the NSCH public-use dataset to facilitate the generation of population-based estimates. These weights were utilized in all analyses presented in our study.

### 2.2. Participants

The merged 2016–2020 NSCH data included a total sample of 25,909 mothers of children aged 0–17 years old with special healthcare needs. After further removing cases with missing information (e.g., insurance type and community type), a total of 17,434 were retained in the final sample. Table 1 provides frequencies of key demographic information of the total sample. Of the 17,434 children, 56.9% of the sample were boys and 43.1% were girls. Participants were predominantly White and non-Hispanic (69.6%). Approximately 16.7% of the sample lived in a rural area. Finally, 35.1% of participants were enrolled in a public insurance plan (e.g., Medicaid).

### 2.3. Measures

#### 2.3.1. Medical Complexity of CSHCN

The medical complexity of CSHCN was assessed with a five-item, mother-report measure based on the Maternal and Child Health Bureau (MCHB) definition of special healthcare needs [37]. The MCHB classification offers a comprehensive assessment of CSHCN by also accounting for the consequences of chronic physical, developmental, or behavioral health conditions that result in health services needs beyond the average child [1]. Participants reported whether the child has been diagnosed with any of the listed chronic health conditions, such as asthma, diabetes, cerebral palsy, and autism. Children diagnosed with one of the designated chronic conditions were classified as CSHCN if they experienced a specific consequence of the diagnosis and the duration was 12 months or longer. The level of complexity was then determined by the number of health consequences the child experiences, including (1) use or need of prescription medication; (2) above average use or need of medical, mental health, or educational services; (3) functional limitations compared with others of the same age; (4) use or need of specialized therapies (OT, PT, speech, etc.); and (5) treatment or counseling for emotional or developmental problems. Selected consequences were totaled resulting in a range of scores from 0 to 5 in which higher scores indicate greater levels of medical complexity.

#### 2.3.2. Healthcare Experiences

Three variables were assessed as separate outcomes of healthcare experiences of mothers—CC, FCC, and SDM. Care coordination (CC) was assessed using the CC subscale of the Measure of Medical Home for Children, an established method within the healthcare system to evaluate the quality of patients’ experiences associated with multiple facets of CC [4]. The measure has also been replicated in studies with community-based (i.e., non-NSCH) samples [39]. This study used one item for effective CC, which was already established by the NSCH using six items regarding the parents’ experiences receiving needed referrals and communication between various providers. The item description was “Did this child receive effective CC?” and the item was assessed using a three-point Likert scale response and recoded as 0 = did not need CC, 1 = did not receive needed CC, and 2 = received needed CC for easier interpretation. Higher scores indicated more effective coordination of the child’s health care services. 

Family-centered care (FCC) was assessed with five items using a four-point Likert scale response ranging from 1 = Always to 4 = Never. Sample questions included “How often did doctors listen carefully to children’s parent?” and “How often did doctors provide information specific to your concerns?” Each item was reverse coded for easier interpretation, such that higher scores indicated higher family-centered care from providers. Responses to each item were then totaled with a range of scores from 5 to 20. Internal consistency for the family-centered care items in the current study was α = 0.92 indicating strong reliability of the measure. 

Shared decision-making (SDM) between parents and providers was assessed with three items using a four-point Likert scale response ranging from 1 = Always to 4 = Never. Three questions included “How often did doctors discuss the range of health care/treatment options?”, “How often did doctors make it easy for parents to raise concerns or disagree with recommendations?”, and “How often did doctors work with parents to decide together health healthcare/treatment options?” Each of the items was reverse-coded for easier interpretation so that higher scores indicate a greater degree of SDM between the provider and the parent. Responses to each item are then totaled with a range of scores from 3 to 12. Internal consistency for the SDM items in the current study was α = 0.91 demonstrating strong reliability of the measure.

#### 2.3.3. Sociodemographic Variables

Child race/ethnicity, community setting, and insurance type were coded as binary variables with a “0” or “1” (i.e., dummy coding) to test for moderation effects. First, the child’s race/ethnicity was assigned as 1 = minority and 0 = White. Community setting was classified as 1 = rural and 0 = urban. Finally, the insurance type was defined as 1 = public and 0 = private. Children’s age (the ages in years), sex (1 = female, 0 = male), and federal poverty level (in categories of FPL by household income and size) were also added as covariates.

### 2.4. Analytical Plan

To test the association between the medical complexity of CSHCN and the healthcare experiences of mothers (H1), we conducted multiple regression analysis in SPSS 28.0. Each healthcare experience outcome was used as a separate outcome. Sociodemographic variables and covariates were added to the regressions. The path coefficients from medical complexity to CC, FCC, and SDM were examined, and significant negative coefficients were expected. The coefficients from these regressions would also reveal the main effects of race/ethnicity, community setting, and insurance type (H2a). 

To test the moderating effects of these demographic factors (H2b), interaction terms between medical complexity (centered), and sociodemographic factors were created to examine their effects on healthcare experiences. Nine moderation models were analyzed in SPSS 28.0 to determine if child race/ethnicity, community setting, and insurance type moderated the relationship between CSHCN complexity and healthcare experiences (i.e., CC, FCC, and SDM). As hypothesized, we expected to see significant interactions suggesting greater vulnerabilities among racially minoritized children living in rural areas and with public insurance.

## 3. Results

### 3.1. Descriptive Statistics

Table 2 shows descriptive statistics and correlations among the variables. From the correlations, the medical complexity of CSHCN was significantly and negatively correlated with CC (r = −0.21, *p* < 0.01), FCC (r = −0.13, *p* < 0.01), and SDM (r = −0.11, *p* < 0.01). These correlations provided preliminary support to H1. CSHCN of minorities with public insurance were related to more medical complexity and negative healthcare experiences (supporting H2a), with community settings yielding mixed findings. With these preliminary findings, we turn to hypothesis testing. 

### 3.2. Hypotheses Testing for the Association between Medical Complexity and Healthcare Experiences (H1)

The results from the multiple regressions are shown in Table 3. Medical complexity of CSHCN was significantly and negatively associated with healthcare experiences including CC (β = −0.21, *p* < 0.01), FCC (β = −0.12, *p* < 0.01), and SDM (β = −0.10, *p* < 0.01), after controlling for sociodemographic factors including race, community, insurance, child sex, age, and federal poverty level.

### 3.3. Hypotheses Testing for the Main and Moderating Effects of Sociodemographic Factors (H2)

To test the main effects of race/ethnicity, community setting, and insurance type (H2a), results are shown in Table 3 above. Minoritized mothers of CSHCN (β = −0.03, *p* < 0.01) who live in rural areas (β = −0.02, *p* < 0.05) reported lower levels of FCC. Federal poverty level was significantly and positively associated with FCC (β = 0.07, p < 0.01) and SDM (β = 0.06, *p* < 0.01). That is, when family incomes exceeded the federal poverty level, they were more likely to report better experiences of FCC and SDM. We also found a significant and positive association between the age of CSHCN and CC (β = 0.02, *p* < 0.05). This indicated that mothers of older CSHCN reported better CC experiences. 

To test the moderating effects (H2b), multiple regressions were conducted with interaction terms created and added to the previous models in Table 4. For CC, the interactions between medical complexity and race (β = 0.05, *p* < 0.05) and between medical complexity and insurance (β = 0.08, *p* < 0.01) were statistically significant. A closer look into the interactions suggested that the negative associations between medical complexity and CC were stronger for White and private insurance than for minorities and public insurance.

For FCC, the interactions between medical complexity and race (β = 0.03, *p* < 0.05) and between medical complexity and insurance (β = 0.06, *p* < 0.01) were statistically significant. For SDM, none of the interactions were significant. Similar to CC models, the interactions suggested that the negative association between medical complexity and FCC was stronger for White and private than for minorities and public insurance. 

## 4. Discussion

Given the public health importance of the well-being of CSHCN and their families, this study sought to fill critical gaps in understanding the healthcare experiences of mothers of CSHCN, particularly those with greater medical complexity. Specifically, this study investigated the association between the medical complexity of CSHCN and the healthcare experiences of mothers, with a focus on CC, FCC, and SDM. Based on family systems theory [18], parenting stress theory [19], and related literature, e.g., [12,22], we hypothesized that the medical complexity of CSHCN would be negatively related to healthcare experiences of mothers of CSHCN (H1). Additionally, we explored the main and moderating effects of sociodemographic factors, including race/ethnicity, community setting, and insurance type on those relationships. Based on a health equity framework [4] and previous research, e.g., [27,28], we hypothesized that minoritized mothers, those with public health insurance, and those in rural areas would be more susceptible to adverse effects of medical complexity (H2). Using a large, nationally representative sample of CSHCN, findings from regression analyses supported H1 and H2a but contradicted H2b.

### 4.1. The Association between Medical Complexity of CSHCN and Healthcare Experiences (H1) 

Consistent with our hypothesis and existing literature, we found that greater medical complexity among CSHCN was significantly associated with more negative healthcare experiences reported by mothers. Specifically, greater medical complexity was associated with lower levels of CC, FCC, and SDM. The findings align with previous research highlighting the adverse impact of medical complexity on various aspects of healthcare delivery and patient experiences [20,22]. As children with greater medical complexity often require more specialized care and services, navigating the healthcare system becomes increasingly challenging for mothers of CSHCN, leading to heightened stress and negative experiences.

In addition to traditional caregiving duties, approximately 17% of caregivers of CSHCN report that they provide over 11 h per day of at-home medical care, exacerbating the burden on family resources, both financially and temporally [40]. This excessive burden is frequently associated with adverse effects on mothers’ mental and physical well-being, including heightened rates of anxiety, depression, and physical health concerns compared to mothers of children in the general population [41,42].

### 4.2. The Main and Moderating Effects of Sociodemographic Factors (H2)

The findings revealed a few important associations that supported our hypothesis. First, minoritized mothers of CSHCN living in rural areas reported lower levels of FCC compared to their counterparts. This highlighted the intersectionality of race/ethnicity and community setting in shaping the healthcare experience, particularly FCC within families and communities. It suggests the presence of disparities that could worsen the difficulties minoritized families encounter in rural areas when caring for their children [27]. Second, rural communities often face challenges such as limited healthcare infrastructure and provider shortages, which could exacerbate disparities in healthcare experiences in FCC for underserved families residing in these areas [32]. Finally, our findings revealed a significant positive association between the federal poverty level and FCC and SDM. This suggested that families with incomes below the federal poverty threshold were more prone to reporting suboptimal experiences of FCC and SDM. Families facing financial constraints may encounter barriers such as lack of insurance coverage, out-of-pocket expenses, and limited access to healthcare providers, all of which can hinder their ability to engage in FCC and SDM processes [43].

In addition to the main effects of sociodemographic factors, we found a few significant interactions between medical complexity and race and insurance type in predicting CC and FCC. However, these findings are contrary to H2b. Specifically, the negative associations between medical complexity and these healthcare experiences were pronounced for White families and those with private insurance compared to minoritized families and those with public insurance. This finding is quite unexpected. One possible reason could be that the measures of healthcare experiences were subjective reports, and mothers of minoritized status and with public insurance may have lower expectations of what’s deemed as “effective” care coordination and quality doctor-parent communication. Regardless, structural inequities play a crucial role in perpetuating disparities in healthcare experiences. Generally speaking, minoritized families and those with public insurance are more likely to encounter systemic barriers, and cultural differences in healthcare delivery [32]. The sociodemographic factors associated with race and insurance type may contribute significantly to health disparities [44]. Our findings highlighted the complexity of healthcare access and utilization and call for future research to examine the nuance of complex relationships. 

### 4.3. Limitations and Future Research

Using large nationally representative data by merging the data of the 2016 through 2020 NSCH to create a nationally representative sample of CSHCN populations, this study contributes to the literature by examining the association between the medical complexity of CSHCN and healthcare experiences and exploring the roles of sociodemographic factors in such association. Despite its strengths, the study has some limitations primarily stemming from the use of secondary data. First, the cross-sectional nature of the NSCH data restricts the ability to establish causal inferences and temporal dynamics between variables. Second, the reliance on self-reported measures, particularly for healthcare experiences, may introduce response bias. Future research could enhance robustness by incorporating data from diverse sources, such as healthcare providers. Third, the correlations between the medical complexity of CSHCN and healthcare experiences, as well as the R-squares in the regression analyses, were fairly small [45]. This suggests that medical complexity explains only a small portion of the variation in healthcare experiences. Many other factors are likely to contribute to the healthcare experiences of mothers of CSHCN. This study serves as an initial step in exploring these associations. Future studies should expand the scope to investigate various influencing factors, providing a more comprehensive understanding of this complex issue. Fourth, this study did not explore potential linking mechanisms between medical complexity, healthcare experience, and maternal well-being, which could be explored in subsequent research by incorporating maternal health variables. Lastly, the impact of national shortages in healthcare professionals, particularly in areas with insufficient availability of providers, is not fully addressed. Future studies could benefit from incorporating data on the health professional shortage area (HPSA) to better understand disparities in healthcare access for CSHCN. Researchers can build upon this study by delving deeper into the mechanisms underlying the relationship between medical complexity, healthcare experiences, and maternal well-being. Longitudinal studies and qualitative research approaches may offer further insights into the dynamics of these relationships over time and across diverse populations.

### 4.4. Implications

Despite the limitations, this study contributes to our understanding of the complex interplay between medical complexity, sociodemographic factors, and healthcare experiences among mothers of CSHCN. By identifying disparities and highlighting areas for intervention, our findings underscore the importance of adopting a comprehensive, family-centered approach to caring for CSHCN. By addressing the complex interplay between medical complexity, healthcare experiences, and sociodemographic factors, stakeholders can collaborate effectively to ensure that all children receive equitable access to high-quality healthcare and support services, regardless of their individual circumstances.

The findings of this study have important implications for healthcare practice and policy aimed at improving the quality of care and outcomes for CSHCN and their families. First, healthcare providers and systems must recognize and address the unique needs of families of CSHCN, particularly those with greater medical complexity. Efforts to enhance CC, promote FCC, and facilitate SDM should be prioritized to mitigate the negative impact of medical complexity on healthcare experiences. Moreover, interventions aimed at reducing healthcare disparities among different demographic groups are crucial. This may involve targeted strategies to improve access to care for underrepresented families, enhance cultural competence among healthcare providers, and address structural barriers to healthcare in rural communities. Additionally, policies that ensure adequate insurance coverage and support for families of CSHCN, regardless of their socioeconomic status or racial/ethnic background, are essential for promoting health equity and improving overall health outcomes.

The implications of this study are manifold, offering insights that can inform various stakeholders engaged in the care of CSHCN. Healthcare providers can leverage these findings to tailor their services effectively, anticipating and addressing the unique challenges faced by CSHCN and their families. By prioritizing aspects such as CC, FCC, and SDM, providers can enhance the quality of care and improve overall outcomes for CSHCN [46]. For policymakers, the study underscores the imperative of targeted interventions to address healthcare disparities among CSHCN and their families. By recognizing and addressing systemic barriers related to sociodemographic factors, policymakers can advocate for policies that promote health equity and ensure equitable access to healthcare services for all children, irrespective of their background or geographic location. Additionally, evaluating the effectiveness of interventions aimed at improving healthcare experiences for CSHCN and their families can guide the development of evidence-based practices. Advocacy groups focused on children’s health and disability rights can leverage the study’s findings to advocate for policies and resources that support the needs of CSHCN and their families. By amplifying the voices of affected families and highlighting areas for improvement in healthcare delivery and access, advocacy groups can drive positive change at the community, state, and national levels.

### 4.5. Conclusions

This study highlights the significant association between the medical complexity of CSHCN and the healthcare experiences of their mothers, focusing on CC, FCC, and SDM. Greater medical complexity correlated with more negative healthcare experience, compounded by sociodemographic disparities affecting minoritized, rural, and lower-income families. These findings emphasize the necessity for targeted interventions to enhance CC, FCC, and SDM, promoting a family-centered approach and addressing systemic inequities. This study calls for continued research and policy efforts to support these underserved populations, reinforming the broader implications of health equity for families with CSHCN. 

## Figures and Tables

**Table 1:** Sample Frequencies of Child Sex, Age, Federal Poverty Level, Cace, Community, and Insurance (*N* = 17,434).

	*N*	%
Sex		
Male	9920	56.9
Female	7514	43.1
Age		
0–3 years	1351	7.7
4–7 years	2759	15.8
8–11 years	4442	25.5
12–14 years	3962	22.7
15–17 years	4920	28.2
Federal Poverty Level		
0–99% FPL	2660	15.3
100–199% FPL	3225	18.5
200–399% FPL	5286	30.3
400% FPL or greater	6263	35.9
Race		
Minorities	5292	30.4
White, non-Hispanic	12,142	69.6
Metropolitan Statistical Area		
Urban	14,517	83.3
Rural	2917	16.7
Insurance Type		
Public	6117	35.1
Private	11,317	64.9

**Table 2:** Descriptive Statistics and Correlations of Model Variables.

Variables	M or % (SD)	1	2	3	4	5	6	7	8	9	10
1. Medical Complexity	2.61 (1.28)	_									
2. Care Coordination	1.61 (0.49)	−0.21 **	_								
3. Family-Centered Care	18.06 (2.77)	−0.13 **	0.50 **	_							
4. Shared Decision-Making	10.74 (1.96)	−0.11 **	0.47 **	0.72 **	_						
5. Race (1 = Minority)	70.5%	0.02 **	−0.02 **	−0.05 **	−0.04 **	_					
6. Community (1 = Rural)	58.3%	−0.01	0.02 **	−0.02 **	−0.01	−0.08 **	_				
7. Insurance (1 = Public)	71%	0.12 **	−0.03 **	−0.07 **	−0.08 **	0.22 **	0.12 **	_			
8. Child sex (1 = Female)	43.6%	−0.08 **	0.01	0.01	−0.01	0.01	0.01	−0.01	_		
9. Child age	11.1 (4.5)	−0.01	0.02 *	0.02 *	0.02 *	−0.05 **	0.02 *	−0.06 **	0.09 **	_	
10. Federal Poverty Level	2.9 (1.01)	−0.11 **	0.03 **	0.10 **	0.09 **	−0.22 **	−0.14 **	−0.60 **	0.01	0.06 **	_

Note. *N* = 17,434. * *p* < 0.05, ** *p* < 0.01.

**Table 3:** Multiple Regressions of Medical Complexity on Healthcare Experiences.

	Care Coordination		Family-Centered Care		Shared Decision-Making	
	β	SE	β	SE	β	SE
CSHCN Complexity	−0.21 **	0.00	−0.12 **	0.02	−0.10 **	0.02
Race (1 = Minority)	−0.01	0.01	−0.03 **	0.05	−0.02	0.05
Community (1 = Rural)	0.02	0.01	−0.02 *	0.06	0.00	0.06
Insurance (1 = Public)	−0.00	0.01	−0.02	0.06	−0.02	0.06
Child Sex (1 = Female)	−0.01	0.01	−0.00	0.04	−0.02	0.04
Child Age	0.02 *	0.00	0.01	0.01	0.02	0.01
Federal Poverty Level	0.00	0.01	0.07 **	0.03	0.06 **	0.02

Note: * *p* < 0.05; ** *p* < 0.01.

**Table 4:** Healthcare Experiences Moderating Effects of Sociodemographic Factors in the Association between Medical Complexity and Healthcare Experiences.

	Care Coordination		Family-Centered Care		Shared Decision-Making	
	β	SE	β	SE	β	SE
CSHCN Complexity	−0.23 **	0.01	−0.13 **	0.02	−0.10 **	0.02
Race (1 = Minority)	−0.05 **	0.02	−0.06 **	0.09	−0.02	0.09
Race × Complexity	0.05 *	0.01	0.03 *	0.04	−0.01	0.01
Child Sex (Female)	−0.01	0.01	−0.01	0.04	−0.03	0.06
Child Age	0.02 *	0.00	0.01	0.01	0.02	0.01
Family Income Level	−0.00	0.01	0.07 **	0.02	0.07 **	0.14 **
$R^{2}$	0.05 **		0.03 **		0.02 **	
CSHCN Complexity	−0.22 **	0.01	−0.12 **	0.02	−0.11 **	0.02
Community (1 = Rural)	−0.01	0.02	−0.03 *	0.11	−0.02	0.11
Community × Complexity	0.03	0.01	0.04	0.04	0.03	0.04
Child Sex (Female)	−0.01	0.01	−0.00	0.04	−0.02	0.04
Child Age	0.02 *	0.00	0.02	0.01	0.02	0.01
Family Income Level	0.01	0.01	0.08 **	0.02	0.08 **	0.02
$R^{2}$	0.05 **		0.02 **		0.02 **	
CSHCN Complexity	−0.24 **	0.02	−0.14 **	0.02	−0.11 **	0.02
Insurance (1 = Public)	−0.08 **	0.01	−0.06 **	0.10	−0.06 *	0.11
Insurance × Complexity	0.08 **	0.02	0.06 **	0.04	0.04	0.03
Child Sex (Female)	−0.01	0.01	−0.00	0.04	−0.02	0.04
Child Age	0.02 *	0.01	0.01	0.01	0.02	0.01
Family Income Level	−0.00	0.01	0.07 **	0.03	0.07	0.02
$R^{2}$	0.05 **		0.02 **		0.02 **	

Note: * *p* < 0.05; ** *p* < 0.01.

## Data Availability

Data used in this study are publicly accessible through the National Survey of Children’s Health website at https://www.childhealthdata.org/help/dataset (accessed on 15 June 2022).
